# Ultrasound-guided superior laryngeal nerve block: a randomized comparison between parasagittal and transverse approach

**DOI:** 10.1186/s12871-024-02612-8

**Published:** 2024-08-03

**Authors:** Tao Shan, Qilian Tan, Dan Wu, Hongguang Bao, Degao Ge, Liu Han, Chuan Su, Yu Ju

**Affiliations:** 1 Department of Anesthesiology, Perioperative and Pain Medicine, Nanjing First Hospital, Nanjing Medical University, Nanjing, Jiangsu Province 210006 China; 2 Department of Anesthesiology, The Second affiliated hospital of Nanjing, University of Chinese Medicine, Nanjing, 210004 China; 3 Center for Global Health, Department of Pathogen Biology and Immunology, Jiangsu Key Laboratory of Pathogen Biology, State Key Lab of Reproductive Medicine, Nanjing Medical University, Nanjing, 211166 China; 4 Wuxi Taihu University, Wuxi, China

**Keywords:** Awake intubation, Parasagittal approach, Superior laryngeal nerve block, Ultrasonography

## Abstract

**Background:**

Different approach ultrasound-guided superior laryngeal nerve block was used to aid awake intubation, but little is known which approach was superior. We aimed to compare the parasagittal and transverse approaches for ultrasound-guided superior laryngeal nerve block in adult patients undergoing awake intubation.

**Methods:**

Fifty patients with awake orotracheal intubation were randomized to receive either a parasagittal or transverse ultrasound-guided superior laryngeal nerve block. The primary outcome was patient’s quality of airway anesthesia grade during insertion of the tube into the trachea. The patients’ tube tolerance score after intubation, total procedure time, mean arterial pressure, heart rate, Ramsay sedation score at each time point, incidence of sore throat both 1 h and 24 h after extubation, and hoarseness before intubation, 1 h and 24 h after extubation were documented.

**Results:**

Patients’ quality of airway anesthesia was significantly better in the parasagittal group than in the transverse group (median grade[IQR], 0 [0–1] vs. 1 [0–1], *P* = 0.036). Patients in the parasagittal approach group had better tube tolerance scores (median score [IQR],1[1–1] vs. 1 [1–1.5], *P* = 0.042) and shorter total procedure time (median time [IQR], 113 s [98.5–125.5] vs. 188 s [149.5–260], *P* < 0.001) than those in the transverse approach group. The incidence of sore throat 24 h after extubation was lower in the parasagittal group (8% vs. 36%, *P* = 0.041). Hoarseness occurred in more than half of the patients in parasagittal group before intubation (72% vs. 40%, *P* = 0.023).

**Conclusions:**

Compared to the transverse approach, the ultrasound-guided parasagittal approach showed improved efficacy in terms of the quality of airway topical anesthesia and shorter total procedure time for superior laryngeal nerve block.

**Trial registration:**

This prospective, randomized controlled trial was approved by the Ethics Committee of Nanjing First Hospital (KY20220425-014) and registered in the Chinese Clinical Trial Registry (19/6/2022, ChiCTR2200061287) prior to patient enrollment. Written informed consent was obtained from all participants in this trial.

## Introduction

Airway management stands as a pivotal procedure in clinical anesthesia, intensive care, and emergency medicine. The presence of a difficult airway significantly amplifies the incidence and severity of associated complications, emerging as a principal contributor to mortality during anesthesia [1, 2]. The technique of awake endotracheal intubation is endorsed by esteemed medical organizations, including the American Society of Anesthesiologists (ASA) and the Difficult Airway Society, as a preferred approach for the management of difficult airway scenarios [3, 4]. To enhance the success of this critical procedure, it is imperative to achieve profound topical anesthesia of the airway, with a particular focus on the laryngeal region. Presently, a spectrum of methodologies exists for attaining this level of anesthesia, prominently featuring the “spray-as-you-go” technique, which is adeptly guided by the precision of a fiberoptic bronchoscope [5] and superior laryngeal nerve (SLN) block assisted with landmarks or ultrasound [6].

The effectiveness of local anesthetic spraying with a fiberoptic bronchoscope relies on the strength of penetration and concentration of the local anesthetic [7]. However, the effects of anesthesia can be significantly limited by laryngeal secretions. Recent research has indicated that an SLN block is more efficient than topical anesthesia in inhibiting the stress response during intubation [8, 9]. However, it can be difficult to achieve excellent topical anesthesia if the anatomical landmarks are obscured, and there is a risk of accidental puncture of the carotid artery [10].

Studies have shown that ultrasonography can improve the technical performance and efficacy of SLN block when placing the transducer transversely [11]. However, locating the limited-sized superior laryngeal nerve or artery using the transverse approach can be challenging, especially in patients with short necks.

Lan et al. [12] presented a new ultrasonic technique for SLN block in cadavers using a parasagittal method. Similarly, Barberet [13] described a parasagittal approach known as the SLN space block. The parasagittal approach allows the identification of all landmarks necessary for the SLN block in a single ultrasonic view. It is reported to be easy to perform even for inexperienced practitioners [12]. However, it is unknown whether this approach is superior to the traditional transverse approaches.

Therefore, we conducted a trial to evaluate the efficacy and safety of ultrasound-guided parasagittal and transverse approach SLN block in patients undergoing awake tracheal intubation. We hypothesed that ultrasound-guided parasagittal approach SLN block may provide superior patient’s quality of airway anesthesia.

## Materials and methods

### Study design and ethics

This was a single-center, randomized, prospective study conducted at Nanjing First Hospital. This study was approved by the ethics committee of Nanjing First Hospital on April 25, 2022 (approval number: KY20220425-01). The study was registered in the Chinese Clinical Trial Registry (registration date: June19, 2022, registration number: ChiCTR2200061287) prior to patient enrollment. Written informed consent was obtained from all participants in this trial.

### Patients and randomization

We recruited patients aged 18–65 years, weighing 45–80 kg, and ASA grade I or II, regardless of sex. The inclusion criteria were as follows: awake intubation characterized by a difficult airway, such as limited movement of the cervical vertebrae, airway insufficiency obstruction, maxillofacial deformity or trauma, small mandibular or mouth opening < 3 cm, Mallampati class III or IV, and full stomach. The patients were scheduled to undergo elective abdominal and/or orthopedic surgeries in the supine position under general anesthesia. Patients with an infection at the puncture site, allergy to local anesthesia, or those who were continuously using anticoagulants prior to surgery were excluded from the trial.

To ensure the random allocation of patients to the two groups, we used a computer-generated sequence of random numbers. The randomization assignment was concealed in opaque envelopes. The allocation of participants into either the parasagittal (PS) or transverse (T) approach group was performed by the same anesthesiologist, who was not involved in the study. All patients underwent superior laryngeal nerve block under ultrasonography. The technique used depended on the assigned group. The PS group underwent superior laryngeal nerve block via the parasagittal approach. The transverse approach targeting the thyrohyoid membrane, superior laryngeal nerve, or artery was used in group T.

### Intervention

#### Preparations before airway topical anesthesia

Before induction, all patients were instructed to abstain from drinking and fasting. Additionally, 0.5 mg of Penehyclidine Hydrochloride was injected intramuscularly 30 min before induction. Routine monitoring, including electrocardiography (ECG), pulse oximetry (SpO_2_), and blood pressure (BP), was performed throughout the procedure. Upper limb venous access was established and a sodium chloride solution was infused. For sedation, midazolam 0.03 mg.kg^-1^ and sufentanil 0.1 µg.kg^-1^were administered intravenously. Radial artery catheterization was then performed to monitor the invasive arterial pressure. 100% oxygen 2 L min^-1^ was administered with a nasal cannula during the procedure.

#### Ultrasound-guided transverse approach SLN block

**Fig. 1:**
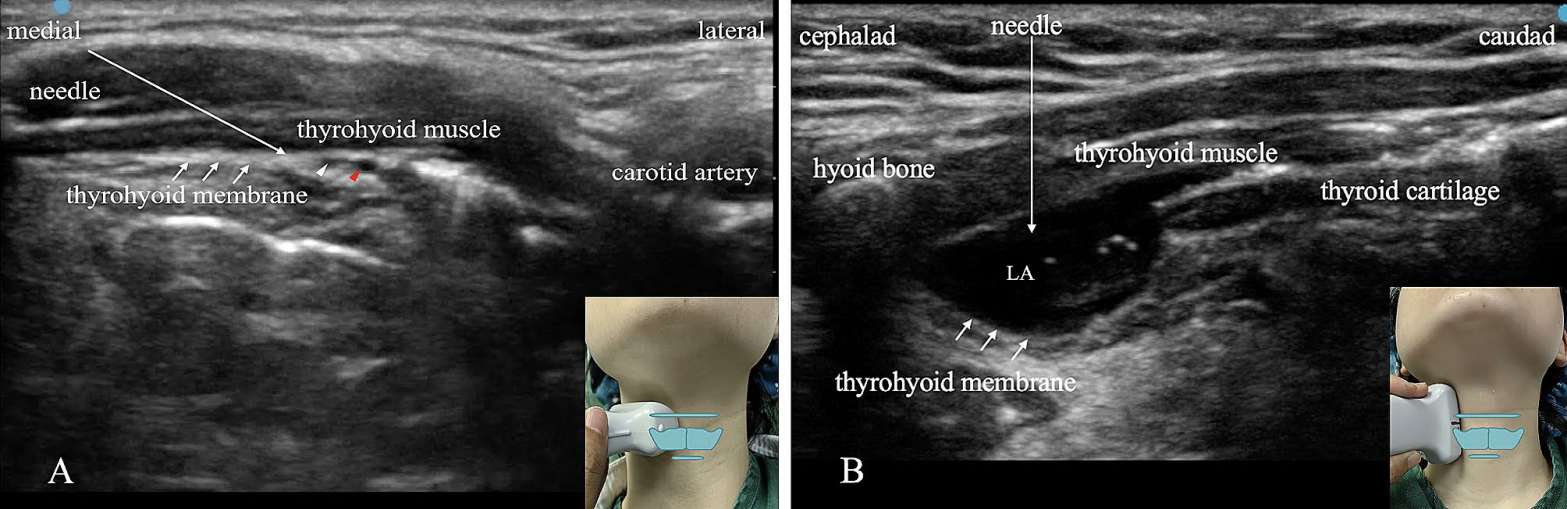
(**A**), Transverse approach ultrasound-guided SLN block with an in-plane technique. Long arrow: block needle; short arrow: thyrohyoid membrane; white short arrow head: superior laryngeal nerve; red short arrow head: superior laryngeal artery. (**B**), Parasagittal approach ultrasound-guided SLN block with an out-of-plane technique. Long arrow: block needle; short arrow: thyrohyoid membrane; LA: local anesthetic

Both groups used a high-frequency 5–13 MHz linear transducer to perform a nerve block (EDGE; SonoSite, USA). In group T, the transverse approach was used [14]. The transducer was positioned transversely between the hyoid bone and the thyroid cartilage to locate the superior laryngeal artery or nerve. If neither of these structures were visible, the thyrohyoid membrane was used as an “anchor” landmark. The block was performed using an in-plane technique from the medial to the lateral direction, and 2% lidocaine (2 mL) was injected bilaterally (Fig. 1A).

#### Ultrasound-guided parasagittal approach SLN block

In the PS group, we placed the transducer longitudinally on the thyroid cartilage and hyoid bone to identify the SLN space. The SLN space, which contains the internal branch of the SLN, characterized with the hyoid bone cephalad, the thyroid cartilage caudad, the thyrohyoid muscle anteriorly, and the thyrohyoid membrane posteriorly. We performed the nerve block with an out-of-plane approach, targeting the injection site medial to the transducer. Once the needle tip was visualized between the thyrohyoid membrane and the thyrohyoid muscle, 2% lidocaine (2 mL) was administered (Fig. 1B).

All patients received topical oral and pharyngeal anesthesia using 2.4% lidocaine spray (Xiangxue Pharmaceutical Co. Ltd, China) administered by an experienced anesthesiologist. The pharyngeal mucosa was meticulously targeted with a lidocaine spray, administered in two brief, one-second bursts. This application was followed by a 5-minute interval before repeating the process, ensuring a total lidocaine dosage of approximately 32 mg per treatment session. Additionally, a transtracheal injection of 3 mL 2% lidocaine between the thyroid and cricoid cartilages was administered to all patients to anesthetize the trachea.

Disposable reinforced endotracheal tube (TUORen Medical Co, Ltd, China) with internal diameters of 7.5 mm and 7.0 mm were utilized for male and female patients respectively and intubated using a fiberoptic bronchoscope (TIC-SD-III, UE Medical Co, Ltd, China) with external diameters 4.2 mm. During the procedure, respiratory depression was considered if SpO_2_ *<* 90%, and the patient was instructed to breathe deeply. Oxygen supplementation was provided via mask ventilation as required. General anesthesia was maintained using intravenous anesthetics. All procedures were performed by the same experienced anesthesiologist and evaluations were conducted by another anesthesiologist who was blinded to the group allocation.

### Outcomes

The primary outcome in this trial was the quality of airway anesthesia assessed on a 5‑point scale [15, 16] by an observer, blinded to the technique of the block, who entered the operating room after the nerve block. The quality of airway anesthesia was graded as follows: 0, no coughing or gagging in response to intubation; 1, mild coughing and/or gagging that did not hinder intubation; 2, moderate coughing and/or gagging that minimally interfered with intubation; 3, severe coughing and/or gagging that made intubation difficult; and 4, very severe coughing and/or gagging that required additional local anesthetic and/or change in technique.

Six secondary outcome measures were specified. (1) Tube tolerance score after intubation [17]. This score assessed the patients’ tolerance to the endotracheal tube after intubation. The scale ranged from 1 to 3, with the following definitions: 1, cooperation; 2, restlessness and mild resistance; and 3, severe resistance requiring immediate general anesthesia. (2) Time taken to identify landmarks (defined as the interval from the placement of the transducer on the skin to the operator’s declaration of completion of skin markings), time taken to administer anesthetic (defined as the interval from needle insertion to the completion of SLN anesthetic injection using the assigned method), and total procedural time (defined as the total time taken to identify landmarks and the time needed to administer the anesthetic). (3) Mean arterial pressure (MAP) and heart rate (HR); these parameters were recorded at different time points, including upon entering the operation room (T_0_), immediately before endotracheal tube insertion (T_1_), immediately after endotracheal tube insertion into the glottis (T_2_), and 5 min after intubation (T_3_). (4) Ramsay sedation score [18], with the sedation level of the patients assessed at each time point. The score ranged from 1 to 6, indicating different levels of sedation: 1, patient anxious, agitated, or restless; 2, patient cooperative, oriented, and tranquil; 3, patient responds to command only; 4, patient asleep, with a brisk response to light glabellar tap or loud auditory stimulus; 5, patient asleep, with a sluggish response to glabellar tap or loud auditory stimulus; and 6, patient shows no response to glabellar tap or loud auditory stimulus. Satisfactory sedation was defined as a score of 2–4, while excessive sedation was defined as a score of 5–6. (5) Incidence of sore throat. The occurrence of sore throat was recorded at 1 h and 24 h after extubation. (6) Incidence of patient hoarseness; assessed before intubation, 1 h after extubation, and 24 h after extubation.

### Sample size calculation and statistical analysis

Sample size calculation was based on the quality of airway anesthesia grade, using preliminary test results from our institution, using PASS 15.0 software (PASS, USA). The difference in the mean score was 0.5, the standard deviation in group PS was 0.67 and in group T was 0.45. With an alpha error of 0.05 and a power of 0.85, 20 patients were required in each group. To account for a potential 20% dropout rate, 50 patients were enrolled in this trial. Although the priori sample size was calculated using a two-sample *t-*test, allowing for unequal variance, the primary outcome was analyzed using the Mann–Whitney *U* test because of the observed distribution of the data after power analysis.

Data analysis was performed using SPSS version 22.0. Normally distributed continuous data are presented as mean (standard deviation) and were analyzed using Student’s t-test. Non-normally distributed continuous data are presented as median (interquartile range) and were analyzed using the Mann–Whitney U test. Categorical data are presented as numbers or percentages and were analyzed using the chi-square test. *P* values *<* 0.05 was considered statistically significant.

## Results

**Fig. 2:**
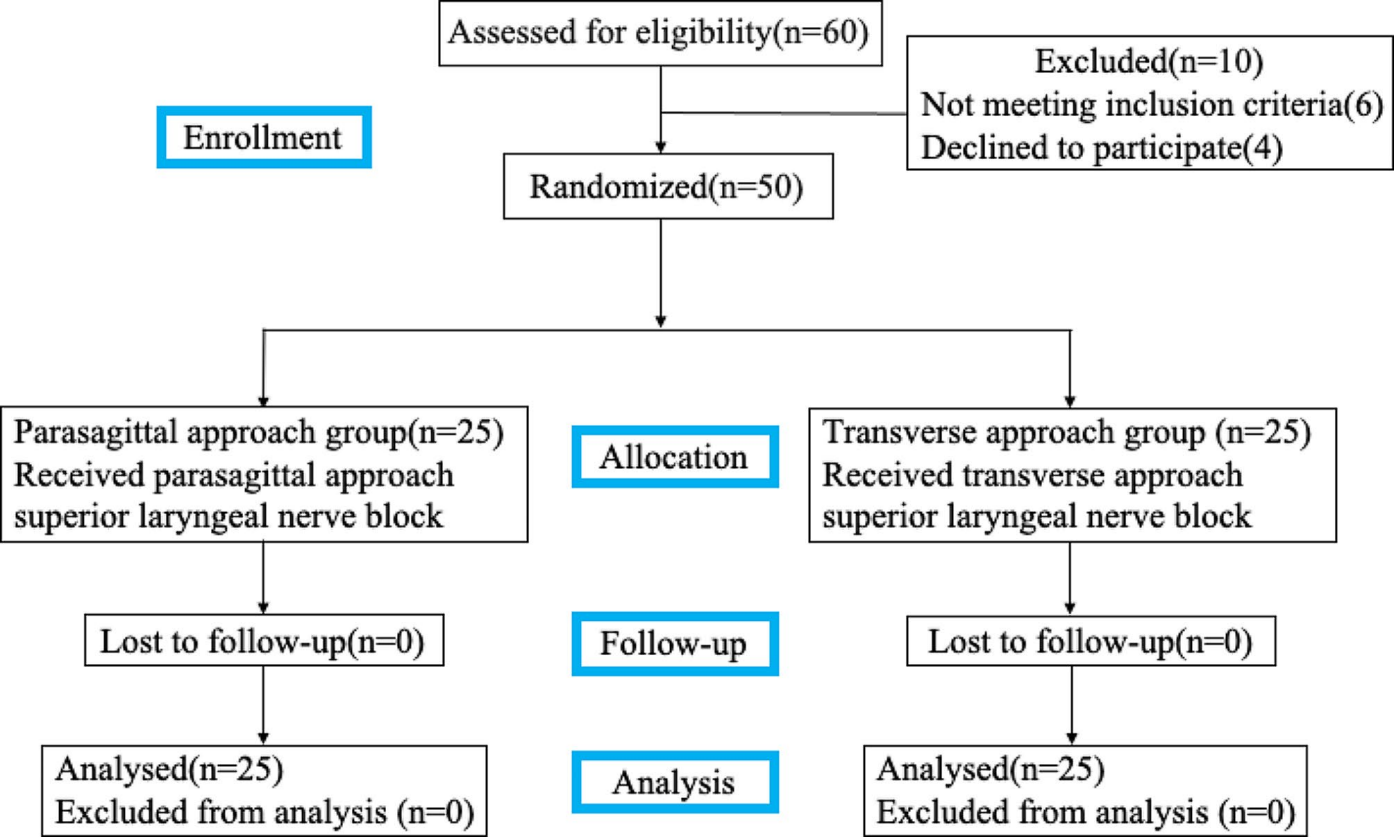
CONSORT flow diagram of trial

We allocated 25 participants to the transverse SLN block group and 25 to the parasagittal SLN block group. A flow diagram of the Consolidated Standards of Reporting Trials (CONSORT) is shown in Fig. 2. The baseline characteristics of the patients, including age, body mass index (BMI), and sex, were comparable between the two groups with no significant differences (*P* > 0.05; Table 1).

**Table 1:** Demographic characteristics

Index	PS group (*n* = 25)	T group (*n* = 25)	t/χ^2^	*P*
Age (years)	46.1 ± 8.1	45.9 ± 9.7	0.08	0.937
Sex (F/M)	15/10	15/10	0.08	0.774
BMI (kg.m^− 2^)	24.6 ± 2.8	23.5 ± 3.6	1.17	0.247
ASA(I/II)	11/14	12/13	0.08	0.777
Airway assessment				
Cervical fracture	18 (72)	17 (68)	0.10	0.758
Coercive spondylitis	4 (16)	5 (20)	0.00	1.000
Mallampati class *IV*	2 (8)	3 (12)	0.00	1.000
Full stomach	1 (4)	0 (0)	-	1.000

*Note* Data are presented as mean ± standard deviation, or number (%). Data presented as mean ± standard deviation were compared using an independent-sample *t*-test or Fisher’s exact *t*-test. Data presented as numbers (%) were compared using Pearson’s chi-square test

*Abbreviations* ASA, American Society of Anesthesiologists; BMI, body mass index

All the patients underwent awake orotracheal intubation. The quality of airway anesthesia, assessed by an observer blinded to the group, showed that patients in the PS group had a lower quality of airway anesthesia grade during insertion of the endotracheal tube over the fiberscope into the trachea (median grade 0, IQR [0–1] vs. 1 [0–1], *P* < 0.05; Table 2). The tube tolerance score after intubation was significantly lower in the PS group than in the T group (median score 1, IQR [1–1]vs. 1 [1–1.5], *P* < 0.05; Table 2). The median total procedural time for performing the SLN block was significantly shorter in the PS group than in the T group (median time 113 s, IQR [98.5–125.5] vs. 188 s [149.5–260], *P* < 0.001; Table 2). The median identification time was 18 s, IQR [16–19] in the parasagittal group and 88 s, IQR [59–132] in the transverse group (*P* < 0.001; Table 2).

**Table 2:** Comparison of quality of airway anesthesia grade, tube tolerance score and time needed between the PS and T groups

Index	PS group (*n* = 25)	T group (*n* = 25)	z	*P*
Quality of airway anesthesia grade	0 [0–1]	1 [0–1]	2.10	0.036
no coughing or gagging, n (%)	18 (72)	11 (44)		
mild coughing and/or gagging, n (%)	6 (24)	10 (40)		
moderate coughing and/or gagging, n (%)	1 (4)	3 (12)		
severe coughing and/or gagging, n (%)	0 (0)	1 (4)		
very severe coughing and/or gagging, n (%)	0 (0)	0 (0)		
Tube tolerance score after intubation	1 [1]	1 [1–1.5]	4.15	0.042
cooperation, restlessness, n (%)	24 (96)	19 (76)		
mild resistance, n (%)	1 (4)	6 (24)		
severe resistance, n (%)	0 (0)	0 (0)		
Performing time (seconds)	96 [81.5–107.5]	98 [88–132]	1.06	0.295
Identifying time (seconds)	18 [16–19]	88 [59–132]	6.01	0.000
Total procedure time (seconds)	113 [98.5–125.5]	188 [149.5–260]	5.12	0.000

*Note* Data are presented as median [IQR], or number (%). Data presented as median [IQR] were compared using the Mann–Whitney U test

The MAP, HR, and Ramsay sedation scores were similar between the two groups, with no significant differences (Table 3).

**Table 3:** Data of hemodynamic profile and Ramsay score during procedure

Index		PS group (*n* = 25)	T group (*n* = 25)	t/z	*P*
MAP (mm Hg)	T_0_	89.1 ± 9.7	90.7 ± 10.7	0.56	0.577
	T_1_	78.8 ± 6.7	81.8 ± 8.5	1.39	0.171
	T_2_	90.3 ± 5.6	91.9 ± 7.6	0.85	0.399
	T_3_	83.6 ± 6.4	85.1 ± 7.5	0.79	0.432
HR (beats/min)	T_0_	71.8 ± 10.9	71.7 ± 8.7	0.01	0.989
	T_1_	67.9 ± 6.0	67.2 ± 6.7	0.36	0.722
	T_2_	72.9 ± 5.5	72.1 ± 7.2	0.44	0.661
	T_3_	69.2 ± 3.8	71.6 ± 8.9	1.21	0.234
Ramsay score	T_0_	3 [2, 3]	3 [2–4]	1.01	0.312
	T_1_	2 [2, 3]	3 [2, 3]	1.54	0.125
	T_2_	3 [2–4]	3 [2–3.5]	0.75	0.452
	T_3_	2 [2, 3]	3 [2–4]	1.77	0.077

*Note* Data are presented as mean ± standard deviation, or median [IQR]. Data presented as mean ± standard deviation were compared using the independent-sample t test. Data presented as median [IQR] were compared using the Mann–Whitney U test

*Abbreviations* HR, heart rate; MAP, mean arterial pressure

The incidence of sore throat 24 h after extubation was significantly lower in the PS group than in the T group (8% vs. 36%, *P* < 0.05; Table 4). Hoarseness occurred in a higher proportion of patients in the PS group than in the T group immediately before intubation (72% vs. 40%, *P* < 0.05; Table 4).

**Table 4:** Comparison of complications between the PS and T groups

	PS group (*n* = 25)	T group (*n* = 25)	χ^2^	*P*
Sore throat, n (%)				
1 h after extubation	3 (12)	5 (20)	0.15	0.700
24 h after extubation	2 (8)	9 (36)	4.20	0.041
Hoarseness, n (%)				
before intubation	18 (72)	10 (40)	5.20	0.023
1 h after extubation	3 (12)	5 (20)	0.11	0.745
24 h after extubation	4 (16)	1 (4)	0.89	0.346

*Note* Data are presented as numbers (%). Data reported as the number of patients (%) were compared using Coontinuity correction of Pearson’s chi-square test

## Discussion

The findings of this trial suggest that the parasagittal approach for the SLN block is superior to the transverse approach in terms of the quality of airway anesthesia during awake orotracheal intubation. The use of the parasagittal approach resulted in an improved quality of topical anesthesia and a shorter total procedure time than the transverse approach guided by ultrasonography.

In previous studies that used the transverse approach, a high-frequency transducer was used to identify the superior laryngeal nerve or artery [19]. However, accurately locating the small-sized landmarks requires rich clinical experience and high-quality ultrasound images. The visualization of these structures is inconsistent and difficult when using the transverse approach. This was consistent with other two trials [20, 21]. Especially in patients with short and thick necks, the gap between the thyroid cartilage and the hyoid bone may be narrow, sometimes only a few millimeters. Therefore, it is difficult to maintain a sharp and steady ultrasound image to visualize related landmarks. These factors collectively lead to poorer quality ultrasonic images and more time spent identifying related structures when performing the SLN block using the transverse approach, potentially resulting in suboptimal efficacy.

In our trial, we noted that the superior performance of the parasagittal approach could be attributed to the enhanced visualization of landmarks using ultrasonography. The hyoid bone, thyroid cartilage, thyrohyoid membrane, and associated muscles are used as anchors in the parasagittal approach [13, 22]. The cephalad hyoid bone and caudad thyroid cartilage [23] served as the primary localization markers. The sonographic appearance of the hyoid bone is distinguished by the crisp acoustic shadow it casts, a feature that facilitates its clear visualization on ultrasound scans. This diagnostic attribute is invaluable for precise hyoid bone identification throughout the procedure. Furthermore, the submandibular gland, situated superior to the hyoid bone, serves as a supplementary landmark, enhancing the accuracy of locating the anatomical structures of interest. Previous studies demonstrated that the submandibular gland can be completely or partially visualized in 3% and 27% of patients, respectively, in the parasagittal plane [6]. This distinctive feature holds profound significance, as it streamlines the process of pinpointing the desired ultrasonic images. By adopting the parasagittal approach, we effectively surmounted the obstacle of elusive identification of the superior laryngeal nerve and artery, thereby rendering the anatomical localization not only more straightforward but also more precise within the scope of our clinical trial. Consequently, this method has markedly enhanced the fidelity of ultrasonic image recognition. These factors ultimately translated into higher-quality airway anesthesia and shorter total procedure time during the parasagittal-approach SLN block.

Sore throat is one of the most common complications of general anesthesia [24, 25]. It has been reported that the SLN block can be employed as an alternative method for preventing or treating pharyngeal pain following general anesthesia [26–29]. In our trial, the incidence of sore throat 24 h after extubation was lower in the parasagittal approach group than in the transverse approach group. Our study was consistent with Zhou’s study [30]. The observed phenomenon could potentially be attributed to the reduced severity of airway mucosal or vocal cord trauma, which can occur as a result of coughing or gagging incidents during the intubation or extubation processes. The parasagittal approach appears to mitigate these risks. Furthermore, it is noteworthy that a substantial proportion—nearly half—of the patients across both study groups reported experiencing hoarseness as a pre-existing condition prior to the intubation procedure. This result was consistent with the findings of Ramkumar [11]. In their study, all patients who received an SLN block had hoarseness postoperatively. This phenomenon can be attributed to the variable and unintentional spread of the injected local anesthetic above the thyrohyoid membrane to the lateral branch of the SLN, resulting in decreased vocal cord tension and subsequent hoarseness. This led us to wonder whether the administration of a local anesthetic under the thyrohyoid membrane can block only the internal branch of the SLN. Further studies are required to test this hypothesis. Overall, these results imply that hoarseness may serve as a simple indicator of a successful SLN block.

Our study had several limitations. First, the trial was conducted by an experienced anesthesiologist, which introduced a potential bias and limited the generalizability of the results to novice practitioners. Second, we did not directly identify the superior laryngeal nerve or artery using the parasagittal approach; thus, potential nerve or artery injuries may occur.

## Conclusion

In conclusion, our trial demonstrated the improved efficacy of the parasagittal approach for ultrasound-guided SLN block in terms of the quality of airway anesthesia, simpler image recognition, and shorter total procedure time compared with the transverse approach, in patients undergoing awake orotracheal intubation. This information may be valuable for training and performing ultrasound-guided SLN block.

## Data Availability

The data supporting the findings of this study are available from the corresponding author upon reasonable request.
